# Clinical Lectures on Pulmonary Consumption

**Published:** 1854-06

**Authors:** 


ART. V.—*Clinical Lectures on Pulmonary Consumption.* By THEOPHILUS THOMPSON, M. D., F. R. S., Fellow of the Royal College of Physicians, London; &c., &c. Philadelphia: Lindsay & Blakiston, 1854; 8vo. pp. 239.

---

These Lectures appeared originally in the London Lancet, and were delivered at the Brompton Hospital for Consumption in 1851.

The most interesting question connected with phthisis, at the present time, is the power of Cod-liver oil to arrest or cure the disease, and a hospital for consumption, where more than six hundred gallons of the remedy are used annually, may be expected to afford some valuable statistics on the subject. The fact that it is administered to such an extent at the hospital is strong evidence of its efficacy. Dr. THOMPSON, as the result of all his observations, places great reliance upon the remedy. He believes that it acts by improving the constitution of the blood, thus improving the general health, restoring the bowels to a natural condition, expanding the pulse, lessening the expectoration, and moderating the night sweats. In many instances, he says, he has found it to supersede the necessity for the use of any other remedy. He has not found the effect of the oil to be, to disorder the bowels, and he has, when given alone, prescribed the eating of a little orange peel, or the taking into the mouth a little table salt, before and after a dose of the remedy, to cover its taste; but he prefers giving the oil combined thus:—

"An ounce and a half of cod-liver oil, four drops of creasote, two drachms of compound tragacanth powder, and four ounces and a half of aniseed water—an ounce to be taken thrice daily."

Dr. THOMPSON has found that cod-liver oil produces its good effects specially in women and children, in whom the proportion of red corpuscles is stated by chemists to be small. He has used it with decided advantage in neuralgia, sciatica, and rheumatism, when those complaints were associated with anemia. There being a pathological condition which it is adapted to correct, it follows that there are cases of phthisis which do not require its use, and in which it is not beneficial; these are cases not marked by anemia. When found appropriate, a persevering use of the oil must be made. Dr. T. relates the case of a patient affected with vomica, in 1848, who was still taking the remedy in 1853. If he omitted it at any time, whatever his diet might be, he lost weight. Once he laid it aside for nine weeks, and in that time his weight fell off ten pounds.

*Cocoa-nut oil* has proved in Dr. THOMPSON's hands an excellent substitute for the oil of the cod-liver. In reference to this vegetable oil he says:—

"The results in the first thirty patients to whom I administered it bear comparison with those obtained in the first thirty-seven patients for whom I prescribed cod liver oil, chiefly in the year 1845. Amongst the patients to whom cocoa-nut oil was given, there were some instances of arrested phthisis, as decided as any I have been accustomed to attribute to the use of cod-liver oil, over which it possesses advantages in reference to economy and palatableness; and it is interesting to remark, that its efficacy was experienced by some who had previously taken cod oil uselessly, and by others who had discontinued it on account of nausea."

The *oil of walnuts* has also been administered in consumption, and the results in some cases have been satisfactory.

There is a growing confidence with the profession in the curableness of consumption. It is now believed not only that the disease may be arrested, but, by some eminent pathologists of large observation, that the tubercular deposit may be removed.

We recommend the lectures of Dr. THOMPSON to our readers. The publishers have issued the work in a style eminently creditable to their taste.

